# The effectiveness of real-time telelactation intervention on breastfeeding outcomes among employed mothers: a systematic review and meta-analysis

**DOI:** 10.1186/s12884-025-07440-3

**Published:** 2025-03-25

**Authors:** Tippawan Iamchareon, Wantana Maneesriwongul

**Affiliations:** Ramathibodi School of Nursing, Faculty of Medicine Ramathibodi Hospital, Mahidol University, 270 Rama VI Rd, Thung Phaya Thai, Ratchathewi, Bangkok, 10400 Thailand

**Keywords:** Breastfeeding outcomes, Employed mothers, Exclusive breastfeeding, Real-time telelactation, Systematic review, Meta-analysis

## Abstract

**Background:**

The global exclusive breastfeeding (EBF) rate during the first six months is < 50%. This rate is particularly low among employed mothers, who may face obstacles in accessing in-person lactation services. Given that telelactation services can increase EBF rates, we conducted this study to assess the effects of real-time telelactation services (vs. usual lactation services) on breastfeeding outcomes among employed mothers.

**Methods:**

The Preferred Reporting Items for Systematic Reviews Meta-Analyses guidelines were followed. Studies published between 2012 and 2023 were identified from the Academic Search Ultimate, Cochrane, CINAHL Complete, Embase, ProQuest, SAGE journals, ScienceDirect, Scopus, Springer Link, Google Scholar, and Thai Journal Online databases. Randomized controlled trials and quasi-experimental studies that met the inclusion criteria were included. The JBI critical appraisal tool was used to assess the studies selected for the systematic review. Categorical data were analyzed using relative risk (RR) with 95% confidence intervals (CIs) and a random-effects model.

**Results:**

Of the 18 studies selected for the review, 13 were included in the meta-analysis with a total of 4,564 participants. Of these, 3,582 were employed mothers. We identified three types of real-time telelactation services based on the activities of the provider and client: proactive, reactive, and mixed services. The results showed that real-time telelactation services had a statistically significant positive effect on the EBF rate during the first six months compared to usual care (Relative risk (RR): 1.31, 95% Confidence interval (CI) [1.10, 1.54]; *p* = 0.002). Proactive and mixed services significantly enhanced the rate of EBF (RR: 1.59, 95% CI [1.23, 2.05]; *p* = 0.0004 and RR: 1.38, 95% CI [1.01, 1.87]; *p* = 0.04, respectively). Reactive services did not significantly affect the EBF rate during the first six months compared to usual care (RR: 0.98, 95% CI [0.93, 1.04]; *p* = 0.54).

**Conclusions:**

Real-time telelactation services delivered by lactation/trained professionals in a proactive or combined proactive/reactive manner (i.e., via scheduled appointments and on demand) were the most effective. These service models should be considered by lactation service providers and healthcare policymakers seeking to increase EBF among the majority of participants who were employed mothers.

**Review registration:**

This review has been registered in the International Prospective Register of Systematic Reviews (PROSPERO) (ID: CRD42023429900).

**Supplementary Information:**

The online version contains supplementary material available at 10.1186/s12884-025-07440-3.

## Background

Breast milk is the best food for newborns [1–3], and breast milk and breastfeeding (BF) have many health benefits for infants and mothers [4–6]. The World Health Organization (WHO) recommends that babies be exclusively breastfed for the first six months and that BF continues alongside the provision of age-appropriate foods for up to two years [7]. The WHO also set the goal of increasing the rate of exclusive breastfeeding (EBF) during the first six months to at least 50% by 2025 [8]. The global rate of EBF was 48% in 2023 [9], and the rates during the first six months are relatively low [9–11], especially among employed mothers [12–16]. According to data from the United Nations Children’s Fund, there is regional variation in the rate of EBF during the first six months, with the rate reaching 51% in Asia, 47% in Africa, 43% in Latin America and the Caribbean, and 26% in North America [9]. A study that involved surveying mothers in 11 European countries found that the rate of EBF at six months varied widely, from 10% in Switzerland to 39% in the Netherlands [17]. In terms of the rates among employed mothers, in developing countries, the rates are reportedly lower in this demographic (10–36%) [11, 14–16, 18]. Maternal employment has been shown to be negatively associated with EBF [19, 20]. In developed countries, 5–32% of employed mothers can continue EBF after returning to work [21]. These relatively low rates are influenced by various factors, including employment-related factors [17, 20]. However, advances in information communication technologies have led to the development of several services, such as telehealth and telelactation services, to increase EBF rates worldwide [22–29]. Telelactation services have been shown to increase access to lactation support, improve BF outcomes, and reduce disparities in BF rates among disadvantaged populations [23, 29].

Two systematic reviews of telelactation topics can be found in the literature [30, 31]. Both reviews do not particularly focus on employed mothers. They generally included employed and non-employed mothers. First, Chua et al. [30] conducted a mixed-studies systematic review by synthesizing data from qualitative and quantitative studies of telelactation services that used real-time audio technology (published between 2007 and 2021). Their results revealed that the included telelactation interventions varied in terms of mode, provider, service time, duration, and frequency; thus, they did not conduct a meta-analysis. Second, Blackmore et al. [31] conducted a systematic review and meta-analysis of interventions that involved virtual telelactation or videoconference consultations versus standard lactation care (published between 2002 and 2021). The BF outcomes were the prevalence of EBF at one, four, and six months. In this meta-analysis, the heterogeneity of the results was very high (*I*^*2*^ = 77–91%) [31], the interventions were not classified according to their type, and studies conducted in Thailand were not included.

Therefore, we aimed to conduct a systematic review and meta-analysis of the current literature to determine the effects of real-time telelactation services on BF among lactating mothers who are employed. To the best of our knowledge, this is one of the first systematic reviews designed to examine the effects of real-time telelactation services on lactating mothers in the context of the working environment. Compared to other reviews, this systematic review had a narrower scope: we focused on the outcomes of telelactation services on EBF during the first six months and on studies in which employed lactating mothers comprised at least 50% of the study cohort. In addition, we classified the telelactation services into three models and compared the effects of each service model on EBF among all lactating mothers and among employed lactating mothers, thereby providing policymakers with valuable insights into the efficacy of different service models and approaches to increase EBF rates worldwide to meet the WHO’s target rate of at least 50% [8].

## Methods

The aims of this study were to (1) describe and categorize the real-time telelactation services used for employed mothers and (2) evaluate the effects of real-time telelactation services versus usual lactation care on BF outcomes.

Our systematic review and meta-analysis were registered with the International Prospective Register of Systematic Reviews (PROSPERO) (ID: CRD42023429900). The Preferred Reporting Items for Systematic Reviews and Meta-Analyses (PRISMA) guidelines were used to conduct and report this study [32]. After searching for and identifying studies that met the inclusion criteria, we extracted the relevant data, appraised its quality, and conducted a meta-analysis. Initially, a meta-analysis of each BF outcome found differences within the data, and the heterogeneity value was > 60% (EBF, BF knowledge, attitude, self-efficacy, and practices). We carried out sensitivity analyses and identified source of heterogeneity. However, there was an inadequate number of studies reporting BF knowledge, attitude, self-efficacy, and practices. We were therefore able to perform subgroup analyses on only two BF outcomes (i.e., EBF and ABF) based on the timing of intervention, types of providers, and types of service models.

### Intervention and comparative condition

Telelactation services delivered in a synchronous manner are referred to as real-time telelactation services. They involve BF support delivered remotely by lactation support staff, such as lactation specialists, trained postpartum nurses, or trained volunteer/peer-support mothers, through telephone calls or video conferencing and the use of various communication devices (e.g., telephones, tablets, and computers) [30, 31, 33–36] and various platforms (e.g., WhatsApp, Line App, Skype, Zoom). In this systematic review, the intervention was the delivery of real-time telelactation services, which allow providers and clients to interact in real time and thus resemble lactation clinic services. We categorized the real-time telelactation services into three models based on the nature of the activities of the provider and client: (1) proactive services, which provide BF education, consultation, or follow-up by appointment; (2) reactive services, which provide lactation services on demand; and (3) mixed services, which provide a combination of proactive and reactive services (i.e., the provider establishes the schedule and number of appointments and simultaneously allows the client to make appointments as needed).

By definition, “usual care” refers to the standard lactation care provided to mothers and infants from birth for six months, based on the recommendations of the WHO [37] and those outlined in the “Ten Steps to Successful Breastfeeding” [38]. Usual care is provided physically on-site (in person) to promote and support lactation. Additionally, all postpartum mothers should receive at least four follow-up visits: within 24 h, between 48 and 72 h, between 7 and 14 days, and during the sixth week postpartum [38]. In this study, the provision of usual care was the comparative condition.

### Outcomes of interest

The outcomes of interest examined in this study were EBF, any breastfeeding (ABF) during the first six months. When EBF occurs, the baby receives breast milk only. No other foods or liquids, including water, are provided during the first six months of life. When ABF occurs, the baby is provided with breast milk and other foods or liquids (including water) [7].

### Search strategy

We conducted a literature search between June and July 2022. We searched the Academic Search Ultimate, Cumulated Index in Nursing and Allied Health Literature (CINAHL Complete), Cochrane, Embase, ProQuest, SAGE journals, Science Direct, Scopus, and Springer Link electronic databases. We also conducted a gray-literature search of the Mahidol University e-Theses database. Advanced searches were conducted using Boolean logic. In addition, we searched the Google Scholar database and manually searched Thai Journals Online, a database of journal articles published in Thailand. To ensure the comprehensiveness of the literature search, the reference lists of the articles included in the existing systematic reviews [30, 31] were also reviewed. Furthermore, we performed another search on June 2023, to ensure that studies published after 2022 were included. The search terms included variations of telemedicine, telehealth, telelactation, BF-related terms, and outcome measures (Supplementary 1). Duplicate articles were removed using EndNote X9 software [39]. The detailed search strategy is shown in Supplementary 1.

### Study selection and criteria

We included randomized controlled trials (RCTs) and quasi-experimental studies that met the following criteria: (1) examined a telemedicine, telehealth, telenursing, or telelactation service delivered in real time (via telephone, video, or an application) compared to a control group that received usual care (in person); (2) evaluated pregnant or postpartum women aged ≥ 18 years who were employed (only studies in which > 50% of the participants were employed mothers were included); (3) provided a detailed description of the intervention; (4) included BF outcomes (EBF rate and ABF rate), and/or other outcomes (BF knowledge, attitude, practices, and self-efficacy); and (5) were written in English or Thai. We excluded nonprimary studies and studies that did not state the percentage of participants who were employed, in which < 50% of the participants were employed, that did not meet the Population Intervention Comparison Outcome Study design (PICOS) criteria, or that had insufficient data available for analysis after three attempts to contact the author. Two independent reviewers (TI and WM) screened the titles and abstracts of the captured articles against the eligibility criteria, after which the full text of the eligible articles was retrieved and independently reviewed.

### Data extraction

Two independent reviewers extracted the relevant information, following the PRISMA checklist, including information on the article’s publication (i.e., authors, publication year, and country), the subjects’ characteristics (i.e., mean age, gravida, parity, and employment status), the intervention (i.e., service mode, service time, duration, provider, and follow-up period), and the outcomes (i.e., effect on EBF and ABF). Discrepancies were resolved through discussion until the reviewers reached a consensus. All of these steps were conducted using the JBI SUMARI tool [40].

### Assessment of risk of bias

The JBI critical appraisal tool for quantitative studies [40] was used to appraise the risk of bias in each included study, namely selection, performance, detection, and attrition bias [41]. The Randomized Controlled Trials Checklist [42] and the Checklist for Quasi-Experimental Studies [43] were used. Two reviewers independently assessed the quality of the studies and used the terms “Yes,” “No,” and “Unclear” to indicate whether bias was present or not. Any disagreements between the reviewers were resolved by rechecking and holding discussions until consensus was achieved.

### Statistical analysis

The collected information was analyzed and synthesized using the JBI SUMARI tool. For dichotomous outcomes (e.g., EBF: yes or no), the relative risk (RR) and 95% confidence interval (CI) were estimated using the random-effects model. Results were considered statistically significant when *p* was < 0.05.

Heterogeneity was assessed using the *I*^*2*^ statistic and the standard χ^2^ test (Q test) with a test level of α = 0.10 [40]. When heterogeneity was present (Q test: *p* < 0.1 or *I*^*2*^ > 50%), the sources of heterogeneity were explored and reduced by sensitivity and subgroup analyses. We performed sensitivity analyses to account for potential publication bias, outlier bias (where a single result significantly impacts the overall estimate), and the statistical weighting of individual effect estimates [44]. Subgroup analysis and publication bias evaluation were performed using RevMan5 software [45].

## Results

**Fig. 1:**
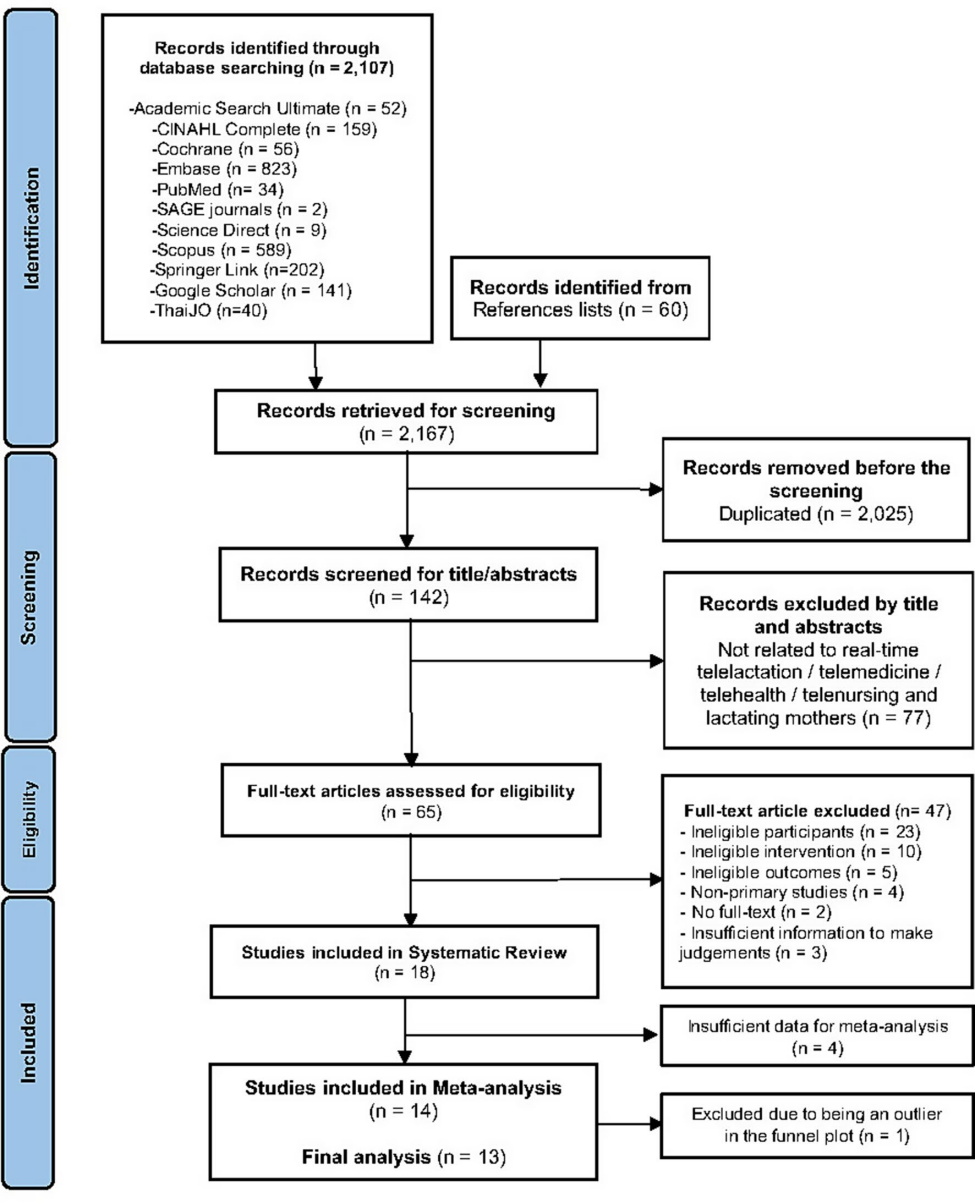
PRISMA flowchart of the study retrieval and selection process

The study selection process is shown in Fig. 1. A total of 11 RCTs and seven quasi-experimental studies were included in the systematic review [46–63]. Of these 18 studies, 14 met the inclusion criteria for meta-analysis [47–51, 53, 54, 57–63]. Data from 4,564 mothers were included in the meta-analysis, 3,582 of whom were employed (78.5%).

### Study characteristics

A total of 18 studies were included in the systematic review [46–63], and the proportion of the participants who were employed mothers varied from 53.7 to 100%. The mean age of the participants ranged from 18 to 35 years. Nine studies were conducted in Asia: two in Hong Kong [48, 50], and seven in Thailand [51–53, 55–57, 59]. Nine studies were conducted with primiparous pregnant women who were in the third trimester of pregnancy and who were followed throughout the postpartum period [46–48, 53, 57–59, 62, 63]. We found that 11 studies provided proactive services [47, 48, 50–52, 55–59, 63], two studies provided reactive services [60, 62], and five studies provided mixed services [46, 49, 53, 54, 61]. The follow-up period ranged from two weeks to six months postpartum. The basic characteristics of the included studies are shown in Table 1.

**Table 1:** The basic characteristics of the included studies (*n* = 18)

1st author/year/ design/	Countries	Characteristics of Participants	*n*, Subjects in Intervention/ Control groups (*n*, Working/ *N* total)	Telelactation Intervention				Outcomes	
				Services activities	Platforms	Providers	Usual care	Follow-up mode/period	BF outcomes
Amin, 2022 [46] Quasi-experiment	Egypt	-Primiparous pregnant women -Mean age: 23.89 ± 1.50 -Employed 88%	60/60 (106/120)	Scheduled online education via Zoom and on-demand consultation via WhatApp support during 2 months	Zoom and WhatsApp	Nurses	Not mentioned	-Interview at 1, and 3 months	1.BF knowledge 2. BF attitude 3. BFSE
Cauble, 2021[47] RCTs	USA	-Primiparous pregnant and Postpartum women -Mean age: 26.2 ± 4.3 -Employed 54%	19/22 (22/41)	Weekly support via telephone call during antenatal (6 sessions)	Acano Audio Conferencing System	IBCLCs	Plus, Usual care	-Email, REDCap survey at 2 weeks, 2, 4, 6 months	1. ABF 2. EBF
Fan, 2022 [48] RCTs	Hong Kong	-Primiparous pregnant women -Mean age: 33.0 ± 4.3 -Employed 82%	18/15 (27/33)	Weekly support and consultation via WhatsApp calls and text messages for 6 months	WhatsApp	Trained peer support	Plus, Usual care	- Telephone F/U at 1, 2, 4, 6 months	1. ABF 2. EBF 3. BF attitude 4. BFSE
Forster, 2019 [49] RCTs	Australia	-Primiparous postpartum women -Mean age: 31.0 ± 5.0 -Employed 96.8%	501/515 (984/1,016)	BF support on days 4–6, then on days 10, weekly in the first 3 months, monthly in 3–6 months, via telephone call, and on mother’s demand support	Telephone	Trained peer volunteer counselors	Plus, Usual care	- Email survey and telephone interview at 6 months	1. ABF 2. EBF
Fu, 2014 [50] RCTs	Hong Kong	-Primiparous postpartum women -Mean age: 30.5 ± 4.5 -Employed 78.5%	255/257 (402/512)	In-hospital support during 24 h and weekly support via telephone call during 4 weeks postpartum	Telephone	Midwife or IBCLCs	Plus, Usual care	- Telephone F/U at 1, 2, 3 months	1. ABF 2. EBF
Iamchareon, 2020 [51] Quasi-experiment	Thailand	-Primiparous postpartum women -Mean age: 23.92 ± 4.05 -Employed 69%	80/77 (108/157)	Onsite providing BF information and practices with BF support via telephone call at 6 weeks, 3 and 6 months	Telephone	Trained nurses	Plus, Usual care	- Telephone F/U at 6 weeks, 3 and 6 months	1. EBF 2. BF knowledge 3. BF practices
Jiewtamai, 2020 [52] Quasi-experiment	Thailand	- Postpartum women -Mean age: 27.61 ± 5.75 -Employed 63.6%	33/33 (42/66)	Onsite providing BF information and practices with scheduled telephone follow up	Telephone	Nurses	Plus, usual care	-Telephone follow up at 6 months	1. BFSE 2. BF practices
Monsaeng, 2016 [53] Quasi-experiment	Thailand	-Pregnant and postpartum women -Age range: 20–30 y. -Employed 76%	29/29 (44/58)	Onsite providing BF information and practices with consultation during service hours and extended hours via telephone Line App for on-demand consultation	Line App	Lactation nurses	Plus, Usual care	- Onsite visit at 1 week, 1, 2–4, and 6 months and telephone F/U at 4 weeks	1. EBF 2. BF knowledge 3. BF practices
Ogaji, 2021 [54] RCTs	Nigeria	-Postpartum women -Mean age: > 30 years -Employed 89%	67/64 (117/131)	- Weekly support in the first two weeks then monthly support via telephone call for 6 months and on mother’s demand call	Telephone	Pediatrician	Plus, Usual care	- Telephone F/U at 1, 2, 3, 4, 5 months and home visit at 6 months	1. EBF
Pengpit, 2022 [55] Quasi-experiment	Thailand	- Postpartum women -Mean age: 29.0 ± 4.72 -Employed 60%	30/30 (36/60)	Onsite providing BF information and practices with scheduled telephone follow up	Telephone	Nurses	Plus, usual care	- Telephone follow up and BF clinic visit at 2 weeks	1. BF knowledge 2. BF attitude 3. BF practices
Phaiboonbunpot, 2015 [56] Quasi-experiment	Thailand	-Primiparous postpartum women -Mean age: 22.8 ± 5.2 -Employed 60%	16/16 (19/32)	Onsite providing BF information and BF practices with scheduled telephone consultation and follow up	Telephone	Nurses	Not mentioned	- Telephone follow up at 2 weeks	1. BF knowledge 2. BF attitude 3. BF practices
Prasitwattanaseree, 2019 [57] RCTs	Thailand	-Primiparous pregnant and postpartum women -Mean age: IG = 27.9 ± 4.6, CG = 27.1 ± 4.7 -Employed 77%	41/42 (64/83)	Onsite providing BF information and practices in ANC with on days 7 support and then monthly support during 3–6 months via telephone call	Telephone	Trained nurses	Plus, Usual care	- Telephone F/U at 7 days, 1, 2, 3, 4, 5, 6 months	1. EBF 2. BFSE 3. BF practices
Puharic, 2020 [58] RCTs	Croatia	-Primiparous pregnant and postpartum women -Age range: 18–35 y. -Employed 75%	129/123 (189/252)	BF Support in antenatal care, then monthly support during the first 3 months postpartum via telephone call	Telephone	Trained nurses	Not mentioned	- Telephone call record at 3, 6 months	1. ABF 2. EBF 3. BF attitude 4. BFSE
Ruenprot, 2023 [59] Quasi-experiment	Thailand	-Primiparous pregnant and postpartum women -Age range: 18–35 y. -Employed 100%	30/30 (60/60)	Online consultation via telephone Line App in the third trimester, 24 h, 72 h, 1 week, and 1 month postpartum with an onsite home visit at 4–8 weeks postpartum	Line App	Trained nurses and peer volunteer	Plus, Usual care	- Home visit at 4–8 weeks and - Telephone line app F/U at 6 months	1. EBF 2. BF knowledge
Seguranyes, 2014 [60] RCTs	Spain	-Postpartum women -Mean age: IG = 31.22 ± 4.71, CG = 31.13 ± 4.76 -Employed 78%	683/718 (1,093/1,401)	On-demand consultation on VDO or telephone call during service hours (8.0 am-8.0 pm)	ASSIR Skype and Hotline phone	Midwives	Plus, Usual care	- Onsite visit, online consulting record, and telephone F/U at 6 weeks	1. EBF
Simonetti, 2012 [61] RCTs	Italy	-Primiparous postpartum women -Mean age: IG = 32.18 ± 3.80, CG = 31.54 ± 3.81 -Employed 69%	55/59 (79/114)	Weekly support via telephone call during 6 weeks postpartum and on mothers’ demand calls	Telephone	Midwives	Plus, Usual care	- Telephone F/U at 1, 3, 5 months	1. EBF
Uscher-Pines, 2020 [62] RCTs	USA	-Pregnant and postpartum women -Mean age: 26.5 ± 5.11 -Employed 55.6%	94/93 (104/187)	On-demand VDO call with 24-hour access and unlimited requests	Pacify Health’s telelactation App	IBCLCs	Plus, Usual care	-Email online survey at 2 weeks, 1, 3 months	1. ABF 2. EBF
Wen, 2020 [63] RCTs	Australia	-Pregnant and postpartum women -Mean age: 32.5 ± 5.0 -Employed 62%	386/385 (478/771)	Every two months support via telephone call during the first 12 months postpartum	Telephone	Trained nurses	Not mentioned	- Telephone F/U at 6 months	1. ABF 2. EBF

**Note. ABF**, Any breastfeeding–the infant receives breast milk, with or without complementary foods or liquids; **ASSIR**, Attention to Sexual and Reproductive Health program; **BF**, Breastfeeding; **BFSE**, Breastfeeding Self-efficacy; **EBF**, Exclusive breastfeeding–the infant receives only breast milk, no other foods, liquids, or water (except for medications, vitamin or mineral supplements); **IBCLCs**, International Board–Certified Lactation Consultations; **Line App**, Freeware app and service for instant messaging, video or voice calling and social networking which is a very popular communication application; **Skype**, a free software that lets people make video and voice calls, send messages, and share files on mobile devices, computers, or tablets; **SMS**, Short Message Service is a text messaging service component of most telephone, Internet and mobile device systems; **WhatsApp**, is an instant messaging and voice-over-Internet. It allows users to send text, voice, and video messages, make voice and video calls, and share photos, documents, users’ locations, and other content; **Zoom**, a communications platform that allows users to connect with video, audio, phone, and chat

### Risk of bias

The issue of bias in the 18 studies included in this systematic review was assessed using the JBI critical appraisal tool [42, 43], and the results are shown in Tables 2 and 3. In two of the RCTs, the randomization method used to assign the participants was unclear and/or the concealment of the allocation to the treatment group was not clearly described. In seven of the studies, there was no mention of whether the study was blinded or the blinding methods. One study did not mention whether the outcome assessment was blinded. In one quasi-experimental study, it was unclear whether the participants included in any of the comparisons received similar treatment and whether appropriate statistical analyses were performed. Another study was ambiguous in terms of the reliability of the outcome measurement. Thus, we concluded that the included studies had a low to moderate risk of bias.

**Table 2:** Critical appraisal of eligible randomized controlled trials

First author and year	Q1	Q2	Q3	Q4	Q5	Q6	Q7	Q8	Q9	Q10	Q11	Q12	Q13
Cauble, 2021 [47]	Y	N	Y	N	N	N	Y	Y	Y	Y	Y	Y	Y
Fan, 2022 [48]	Y	Y	Y	N	N	N	Y	Y	Y	Y	Y	Y	Y
Forster, 2019 [49]	Y	Y	Y	N	N	N	Y	Y	Y	Y	Y	Y	Y
Fu, 2014 [50]	Y	Y	Y	N	N	N	Y	Y	Y	Y	Y	Y	Y
Ogaji, 2020 [54]	Y	Y	Y	Y	Y	N	Y	Y	Y	Y	Y	Y	Y
Prasitwattanaseree, 2019 [57]	Y	Y	Y	U	U	U	Y	Y	Y	Y	Y	Y	Y
Puharic, 2020 [58]	U	Y	Y	U	U	U	Y	Y	Y	Y	Y	Y	Y
Seguranyes, 2014 [60]	Y	Y	Y	Y	Y	U	Y	Y	Y	Y	Y	Y	Y
Simonetti, 2012 [61]	U	U	Y	U	U	U	Y	Y	Y	Y	Y	Y	Y
Uscher-Pines, 2020 [62]	Y	Y	Y	Y	Y	Y	Y	Y	Y	Y	Y	Y	Y
Wen, 2020 [63]	Y	Y	Y	Y	Y	Y	Y	Y	Y	Y	Y	Y	Y

**Table 3:** Critical appraisal of eligible quasi-experimental study

First author and year	Q1	Q2	Q3	Q4	Q5	Q6	Q7	Q8	Q9
Amin, 2022 [46]	Y	Y	U	Y	Y	Y	Y	Y	Y
Iamchareon, 2020 [51]	Y	Y	U	Y	Y	Y	Y	Y	U
Jiewtamai, 2020 [52]	Y	Y	Y	Y	Y	Y	Y	Y	Y
Monsaeng, 2016 [53]	Y	Y	Y	Y	Y	Y	Y	Y	Y
Pengpit, 2022 [55]	Y	Y	Y	Y	Y	Y	Y	Y	Y
Phaiboonbunpot, 2015 [56]	Y	Y	Y	Y	Y	Y	Y	Y	Y
Ruenprot, 2023 [59]	Y	Y	Y	Y	Y	Y	Y	U	Y

### Service models utilized in the included studies

Regarding the service models examined in the studies, 11 studies focused on the proactive service model [47, 48, 50–52, 55–59, 63]. The providers proactively set the activities and schedules and contacted the mothers. The clients received BF information, participated in consultations, or attended follow-up appointments, in addition to receiving usual care. The intervention was initiated during the antenatal period [46–48, 53, 57–59, 62, 63]. Two studies focused on the reactive service model [60, 62]. Mothers could seek support via telephone or video calls during service hours [60] or at any time (24 h) [62]. Five studies focused on the mixed-service model. In these studies, services were delivered during scheduled appointments, consultations, or follow-ups, and on-demand support was also available [46, 49, 53, 54, 61]. The basic characteristics of the real-time telelactation service models are presented in Table 4.

**Table 4:** Characteristics of the real-time telelactation service models

Characteristics	I Proactive service	II Reactive service	III Mixed service
Number of studies included in systematic review (in meta-analysis)	11 studies [47, 48, 50–52, 55–59, 63] (7 studies) [47, 48, 50, 51, 57, 59, 63]	2 studies [60, 62] (2 studies) [60, 62]	5 studies [46, 49, 53, 54, 61] (4 studies) [49, 53, 54, 61]
Communication mode	-Group / Individual -Telephone / WhatsApp / Line App call support -Face to face	- Individual - Telephone call / VDO call support / Skype	-Individual -Telephone / Line App / VDO call support / Zoom meeting / SMS -Face to face
Service mode	-Online appointment service -Onsite appointment service -Consultation/ information/follow-up calls set by providers	-Online on-demand service - Consultation requested by participants	-Online appointment / on-demand service -Onsite appointment service -Consultation/information/ follow-up set by providers -Consultation requested by participants
Service time	-During service hours	-Open 24-hours -During service hours (8 am-8 pm)	-During service hours and extended hours -Telephone call timing was convenient for both parties.
Providers	- Lactation / trained professionals / IBCLCs -Trained-mothers volunteer	- Lactation / trained professionals / IBCLCs	- Lactation/trained professionals -Trained-mothers volunteer

**Note. IBCLCs**: International Board-Certified Lactation Consultations; **Line App**: Freeware apps and services for instant messaging, video or voice calling and social networking are very popular communication applications; **Skype**: a free software that lets people make video and voice calls, send messages, and share files on mobile devices, computers, or tablets; **SMS**: Short Message Service is a text messaging service component of most telephone, Internet and mobile device systems; **WhatsApp**: is an instant messaging and voice-over-Internet. It allows users to send text, voice, and video messages, make voice and video calls, and share photos, documents, users’ locations, and other content; **Zoom**: a communications platform that allows users to connect with video, audio, phone, and chat

### Effect of real-time telelactation services on EBF during the first six months

**Fig. 2:**
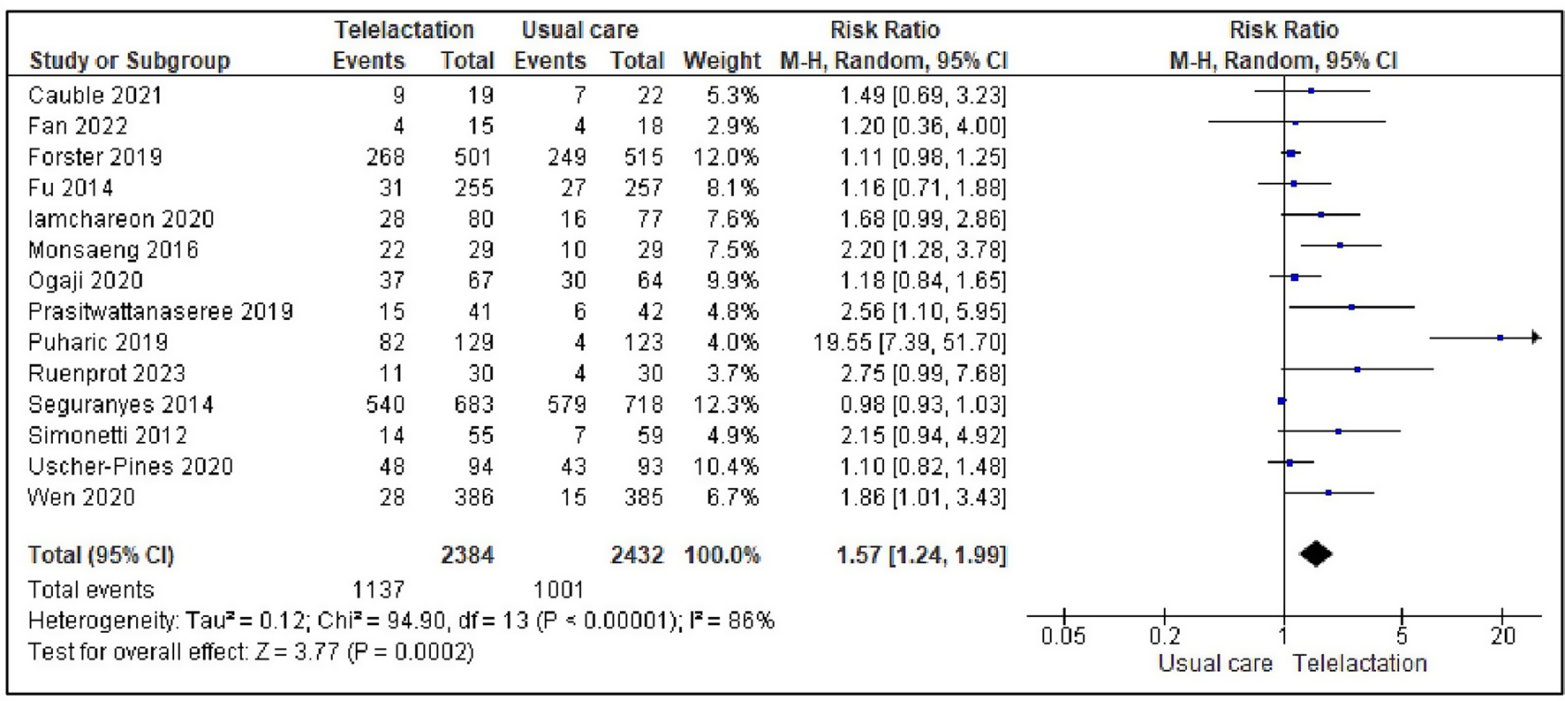
Forest plot of pooled effect size of real-time telelactation on EBF (14 studies)

**Fig. 3:**
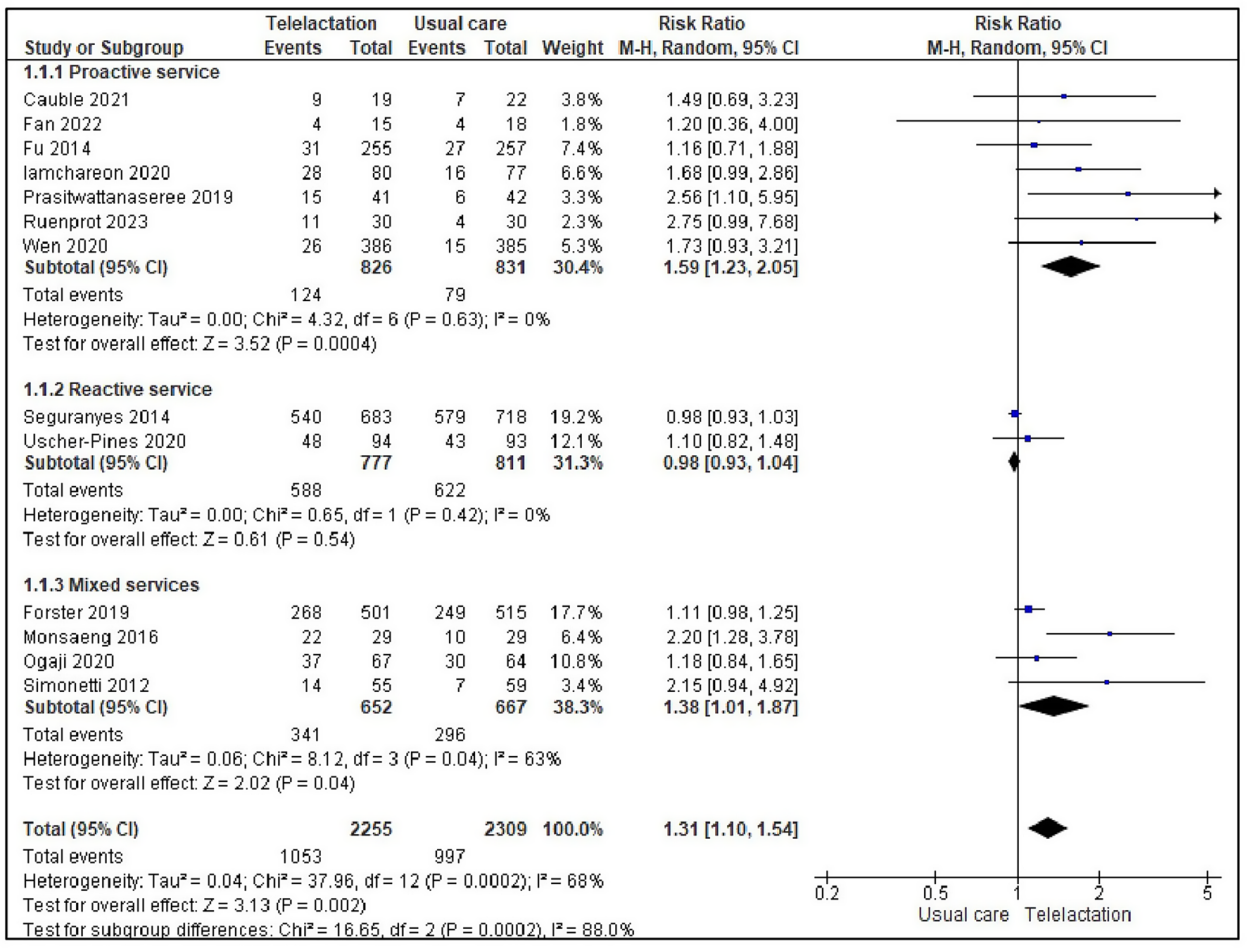
Forest plot of pooled effect size of real-time telelactation on EBF (13 studies)

A preliminary analysis of the data extracted from the 14 studies included in the meta-analysis revealed that real-time telelactation services had a significant positive effect on EBF during the first six months postpartum compared to usual care (RR: 1.57, 95% CI [1.24, 1.99], *p* = 0.0002). However, substantial heterogeneity was observed across the studies (*I*^*2*^ = 86%; Fig. 2). Thus, we explored the potential causes of this heterogeneity using sensitivity analysis and subgroup analysis. The sensitivity analysis was performed by removing studies one by one and examining the resultant funnel plot. One study [58] was determined to be a potential outlier and was subsequently excluded. Excluding this study reduced the heterogeneity (*I*^*2*^ = 68%). The pooled analysis across the remaining 13 studies showed a statistically significant positive effect of real-time telelactation services on EBF (RR: 1.31, 95% CI [1.10, 1.54], *p* = 0.002). This indicated that lactating mothers who received real-time telelactation services were 31% more likely to exclusively BF their infants for the first six months than those who received the usual care (Fig. 3).

**Fig. 4:**
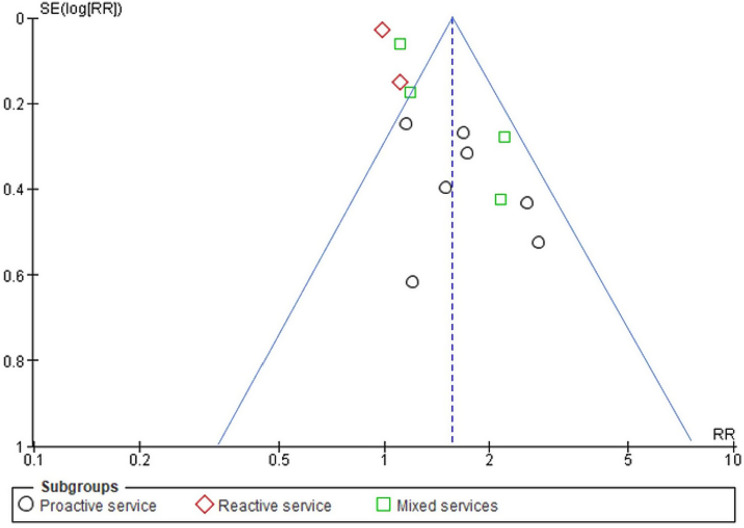
Funnel plot generated during the publication bias analysis of the 13 included studies

The results of the subgroup analysis, which was based on the timing of the intervention (intervention initiated during the third trimester vs. the postpartum period), types of providers, and types of service models, showed that the following factors may have increased the heterogeneity: starting the intervention during the postpartum period (*I*^*2*^ = 63%) and using the mixed-services model (*I*^*2*^ *=* 63%) (Supplementary 2). As shown in the funnel plot generated using the data of the 13 included studies (Fig. 4), no publication bias was detected.

### Proactive service model

Among the studies that focused on the proactive service model, EBF during the first six months postpartum was reported in seven [47, 48, 50, 51, 57, 59, 63]. In these studies, in which the providers scheduled consultations and follow-up appointments, the intervention was shown to have a significant positive effect on EBF (RR: 1.59, 95% CI [1.23, 2.05]; *p* = 0.0004), with no heterogeneity (*I*^*2*^ = 0%). Mothers who received proactively delivered real-time telelactation services were 59% more likely to exclusively breastfeed their babies than those who received only usual care. This result suggests that providing telelactation services in a structured and proactive manner may effectively promote EBF among employed mothers (Fig. 3).

### Reactive service model

In two studies that focused on the reactive service model [60, 62], EBF was found to occur during the first six months. In this subgroup, in which consultations were initiated by the participants as needed, the effect of the intervention on EBF was not statistically significant (RR: 0.98, 95% CI [0.93, 1.04]; *p* = 0.54), and there was no heterogeneity (*I*^*2*^ = 0%). This indicates that providing services in a reactive manner may be less effective than providing services in a proactive manner in terms of increasing the rate of EBF (Fig. 3).

### Mixed services model

In the four studies that focused on the mixed-service model [49, 53, 54, 61], EBF was found to occur during the first six months. The combination of provider- and client-driven consultations had a statistically significant positive effect on EBF (RR: 1.38, 95% CI [1.01, 1.87]; *p* = 0.04). Mothers who received services delivered via the mixed-service model were 38% more likely to continue EBF during the first six months than those who received the usual care. However, moderate heterogeneity was observed in this subgroup (*I*^*2*^ = 63%), which suggested that the effectiveness of the mixed service model may depend on the specific implementation and context [40] (Fig. 3).

### Effect of real-time telelactation services on ABF during the first six months

**Fig. 5:**
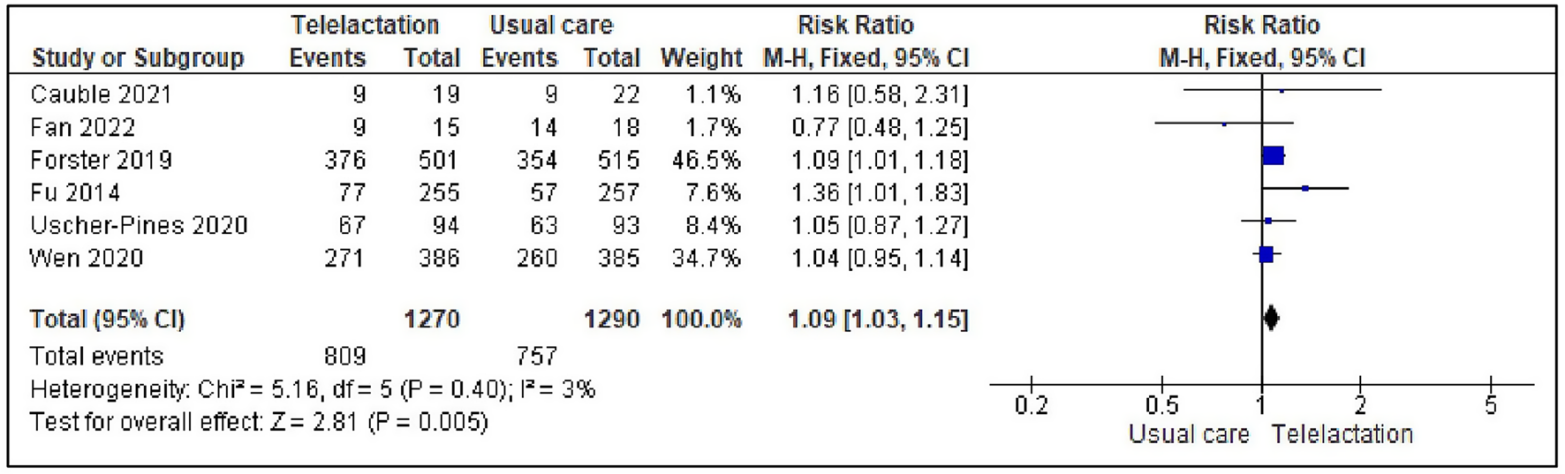
Forest plot of pooled effect size of real-time telelactation services on ABF (6 studies)

Six of the studies [47–50, 62, 63] reported the effect of the intervention on ABF during the first six months. The fixed-effects model analysis showed that receiving real-time telelactation services significantly increased the rate of ABF during the first six months compared to receiving usual care (RR: 1.09, 95% CI [1.03, 1.15]; *p* = 0.005), with low heterogeneity (*I*^*2*^ = 3%). This indicated that the mothers who received telelactation services were 9% more likely to engage in BF compared to those who received the usual care. The outcome of a sensitivity test showed that the results were stable. The forest plot generated from these results is shown in Fig. 5.

## Discussion

In this study, we aimed to evaluate the effect of real-time telelactation services on EBF among the majority of participants who were employed mothers compared to usual care. To achieve this aim, we performed a meta-analysis in which we examined 10 RCTs and three quasi-experimental studies that encompassed 4,564 mothers in which 3,582 were employed mothers. We focused on real-time telelactation services (delivered through telephone calls or videoconferencing) and lactating mothers particularly employed. In addition, we categorized the services into three service models (proactive, reactive, and mixed services) for a deeper analysis of the effects of the services on EBF.

The results suggest that delivering real-time telelactation services, particularly those delivered in a proactive manner, can effectively increase EBF rates among employed mothers. Our results align with those of Blackmore et al. [31], who undertook a systematic review and meta-analysis to investigate the effects of virtual lactation support on BF.

Our results also indicate that delivering telelactation services in a proactive manner significantly enhances the rate of EBF among employed mothers. This may be because appointments can be scheduled at times and on dates that are convenient for them. In their mixed-studies systematic review, Chua et al. [30] found that services offered in a proactive manner fostered stronger relationships between healthcare providers and mothers than services offered in a reactive manner. Proactive service sessions are typically longer, arranged in advance, and conducted with a familiar provider. We found that proactive telelactation services, such as follow-up telephone or video calls and counseling sessions, improved the mother’s morale and confidence in BF. These services also support the effective management of BF barriers or challenges [47, 57, 59]. However, an adequate number of healthcare providers with proper training and supporting resources are needed to offer proactive telelactation services [64].

Reactive services are typically offered either 24 hours a day [62] or between set operating hours (e.g., 8:00 a.m. to 8:00 p.m.) [60], and mothers can call or video conference with providers based on their needs. Although such services are available on demand for urgent needs, they may not allow continuity of care from a familiar provider. This may reduce the rate of EBF among employed mothers and thus be a possible reason for reactive services being less effective than proactive services. Another challenge associated with reactive services is their availability. Mothers may find it difficult to access services at a convenient time. It has been shown that most mothers seek consultations after 6:00 p.m., typically after they have completed their work duties [23, 65]. In addition, these consultations are often brief, focusing on urgent problems or needs, and once the issue is resolved, there is little need for further contact. This could lead to a lack of follow-up and result in reduced continuity of BF support, which may reduce the rate of EBF [33, 34].

The mixed-service model, which is a combination of proactive and reactive service models, shows promise as a tool for the delivery of real-time telelactation services. Delivering services using the mixed-service model was found to increase EBF rates in a manner similar to using the proactive service model; however, the increase was only marginal [49, 54, 61], and there were associated increases in labor intensity and costs [29, 64, 65]. Hence, it may be less feasible to apply the mixed-service model in resource-limited settings.

Real-time telelactation services have been shown to enhance regular and ongoing contact between the clients and providers, and to significantly boost EBF rates [28, 31, 33]. In addition, the flexible nature of telelactation services (including helplines, virtual consultations, and asynchronous messaging) helps employed mothers access lactation support and education at times that fit their schedules. Hence, providing more telelactation services could boost access to lactation support, improve BF outcomes, and address the barriers that limit in-person lactation clinic visits, especially among employed lactating mothers [19, 29, 53, 65]. Nevertheless, there are some individual and institutional constraints that prevent the optimal delivery of telelactation services. For example, the use of video calls has also been identified as a barrier, particularly for mothers who are concerned about their privacy during consultations, as breastfeeding issues are often sensitive [64, 65]. In resource-limited settings, healthcare providers often lack the resources and/or training required to offer comprehensive BF support [34, 64]. However, it is worth noting that a 2017 Cochrane review [66] of BF support found that additional support could reduce the likelihood of early cessation of BF, regardless of the person delivering it [31, 66], which suggests that the frequency of contact may be more important than the provider’s expertise in terms of prolonging the duration of EBF [66]. Other challenges related to service accessibility and the need for sustained ongoing support must also be addressed to enhance the effectiveness of real-time telelactation services [67].

The analyzed studies displayed moderate variation, suggesting that the occurrence of BF was affected by the service model, provider, and/or service activities, duration, and/or frequency. The heterogeneity observed across the studies emphasizes the need to consider possible sources of heterogeneity, such as service intensity, providers’ expertise, and clients’ characteristics when interpreting results and implementing telelactation services. Further studies are needed to determine the optimal balance between provider- and client-initiated support.

As mentioned earlier, despite the well-established benefits of BF, EBF rates remain suboptimal, particularly among employed mothers. Our findings indicate that providing real-time telelactation services is an effective strategy for improving EBF rates among employed mothers. Our findings also suggest that delivering telelactation services in a proactive manner may be a particularly effective strategy for supporting employed mothers and should be considered by lactation service providers and healthcare policymakers seeking to increase EBF among employed mothers. Furthermore, our findings highlight the need for studies that focus on lactating mothers who are employed, as their situations are markedly different from those who are not employed.

### Strengths and limitations

Given that 78.5% (3,582/4,564) of the participants in the 13 included studies were employed mothers, a strength of this study is that its findings can be used to develop strategies for increasing EBF within this specific demographic. Another strength of this study is that we categorized telelactation services into three models. This will support the tailoring of such services to the needs of employed mothers.

However, this study has some limitations. First, we included only studies published in English or Thai. This may have led to relevant studies published in other languages being excluded, which potentially impacts the generalizability and breadth of the findings. Second, the moderate heterogeneity detected in this study may have influenced the results. A network meta-analysis should be conducted to test the effectiveness of the different telelactation service models. Lastly, the approximately 20% of non-working mothers may dilute the overall findings.

## Conclusions

The findings of this study show that real-time telelactation services are effective interventions for enhancing EBF among lactating mothers particularly those who were employed, when delivered through proactive and mixed-service models. Lactation service providers and healthcare policymakers seeking to promote EBF among employed mothers should consider offering more real-time telelactation services in a proactive or combined proactive/reactive manner.

## Electronic supplementary material

Below is the link to the electronic supplementary material.

Supplementary Material 1

Supplementary Material 2

## Data Availability

All data generated or analyzed during this study are included in this published article and its supplementary information files.

## Abbreviations

ABF: Any Breastfeeding
BF: Breastfeeding
CI: Confidence Interval
EBF: Exclusive Breastfeeding
PICOS: Population, Intervention, Comparison, Outcome, Study design
PRISMA: Preferred Reporting Items for Systematic Reviews and Meta-Analyses
RCTs: Randomized Controlled Trials
RR: Relative risk
WHO: World Health Organization
