# Supplementary material for: The effectiveness of real-time telelactation intervention on breastfeeding outcomes among employed mothers: a systematic review and meta-analysis
Source: BMC Pregnancy Childbirth. 2025 Mar 25;25:341. doi: 10.1186/s12884-025-07440-3 (PMC11934809; doi:10.1186/s12884-025-07440-3)
Supplement: Supplementary file 1 — Supplementary Material 1 [file 12884_2025_7440_MOESM1_ESM.pdf]

Supplementary 1 Completed search strategy

Search conducted on **PubMed** from 2012 to 2022 (5/06/2022)

PICO	Search Terms	Numbers of studies
POPULATION	#1 Pregnan*	362,629
	#2 Pregnant women	65,002
	#3 Postpartum mothers	504
	#4 Mother	135,845
	#5 Postpartum Period	16,703
	#6 Postpartum	57,058
	#7 Postpartum mother	63
	#8 Postpartum Women	3,867
	#9 Puerperium	35,922
	#10 Working women	1,461
	#11 Working mother	67
	#12 Working mothers	372
	#13 Working mom	1
	#14 Employed mother	23
	#15 Employed mother*	183
	#16 #1OR #2 OR #3 OR #4 OR #5 OR #6 OR #7 OR #8 OR #9 OR #10 OR #11 OR #12 OR #13 OR #14 OR #15	454,851
INTERVENTION	#17 Real-time	260,178
	#18 Online	225,807
	#19 Tele-lactation	5
	#20 Telelactation	20
	#21 Telehealth	45,703
	#22 Telemedicine	40,893
	#23 Remote lactation	111
	#24 Mobile lactation	1
	#25 Mobile breastfeeding	246
	#26 mHealth	47,842
	#27 Telephone support	577
	#28 Telephone call	782
	#29 Helpline	782
	#30 Hotline	1,474
	#31 Virtual lactation	96
	#32 Virtual breastfeeding service*	25
	#33 Videoconference	4,124
	#34 Video call	305

PICO	Search Terms	Numbers of studies
	#35 #17 OR #18 OR #19 OR #20 OR #21 OR #22 OR #23 OR #24 OR #25 OR #26 OR #27 OR #28 OR #29 OR #30 OR #31 OR #32 OR #33 OR #34	529,935
COMPARISON	#36 Usual care	17,390
	#37 Care Standard	220
	#38 Care Standards	32,581
	#39 Standards of Care	3,197
	#40 Normal care	139
	#41 Traditional care	490
	#42 Routine care	6,871
	#43 #36 OR #37 OR #38 OR #39 OR #40 OR #41 OR #42	60,126
OUTCOME	#44 Exclusive breastfeeding	3,921
	#45 Any breastfeeding	404
	#46 Breastfed	23,132
	#47 Rate of exclusive breastfeeding	2,089
	#48 Breastfeeding rate	302
	#49 Duration of breastfeeding	1,016
	#50 Breastfeeding duration	1,553
	#51 Breastfeeding skill	698
	#52 Breastfeeding efficacy	17
	#53 Breastfeeding practice	292
	#54 Breastfeeding Self-efficacy	340
	#55 Breastfeeding knowledge	262
	#56 #44 OR #45 OR #46 OR #47 OR #48 OR #49 OR #50 OR # 51 OR #52 OR #53 OR #54 OR #55	24,362
	#57 #16 AND #35 AND #43 AND #56	34

Initial keyword to be used will be: Pregnan* or Pregnant women or Postpartum mothers or Mother or Postpartum Period or Postpartum or Postpartum mother or Postpartum Women or Puerperium or Working women or Working mother or Working mothers or Working mom or Employed mother or Employed mother* AND Real-time or Online or Tele-lactation or Telelactation or Telehealth or Telemedicine or Remote lactation or Mobile lactation or Mobile breastfeeding or mHealth or Telephone support or Telephone call or Helpline or Hotline or Virtual lactation or Virtual breastfeeding service* or Videoconference* or Video call AND Usual care or Care Standard or Care Standards or Standards of Care or Normal care or Traditional care or Routine care AND Exclusive breastfeeding or Any breastfeeding or Breastfed

or Rate of exclusive breastfeeding or Breastfeeding rate or Duration of breastfeeding or Breastfeeding duration or Breastfeeding skill or Breastfeeding efficacy or Breastfeeding practice or Breastfeeding Self-efficacy or Breastfeeding knowledge.

Limit: 1. Year 2012-2022 2. English or Thai

Search conducted on **Academic Search Ultimate** from 2012 to 2022 (7/06/2022)

PICO	Search Terms	Numbers of studies
Participants	Pregnan* OR "Pregnant women" OR "Postpartum mothers" OR Mother OR "Postpartum Period" OR Postpartum OR "Postpartum mother" OR "Postpartum women" OR "Puerperium OR "Working women" OR "Working mother" OR "Working mothers" OR "Working mom" OR "Employed mother" OR "Employed mother*"	1,122,336
Intervention	Real-time OR Online OR Tele-lactation OR Telelactation OR Telehealth OR Telemedicine OR “Remote lactation” OR “Mobile lactation” OR “Mobile breastfeeding” OR mHealth OR “Telephone support” OR “Telephone call” OR Helpline OR Hotline OR “Virtual lactation” OR “Virtual breastfeeding service*” OR Videoconference* OR “Video call”	858,899
Comparison	“Usual care” OR “Care Standard” OR “Standards Care” OR “Normal care” OR “Traditional care” OR “Routine care”	13,729
Outcomes	“Exclusive breastfeeding” OR “Any breastfeeding” OR Breastfed OR “Rate of exclusive breastfeeding” OR “Breastfeeding rate” OR “Duration of breastfeeding” OR “Breastfeeding duration” OR “Breastfeeding skill*” OR “Breastfeeding efficacy” OR “Breastfeeding practice” OR “Breastfeeding Self-efficacy” OR “Breastfeeding knowledge”	10,781
	P AND I AND C AND O	52

Limitation: 1. Year: 2012-2022 2. Language: English or Thai
3. Articles Type: Academic Journals

Search conducted on **SAGE Journal** from 2012 to 2022 (7/06/2022)

PICO	Search Terms	Numbers of studies
Participants	Pregnancy OR "Pregnant women" OR "Postpartum mothers" OR Mother OR "Postpartum Period" OR Postpartum OR "Postpartum mother" OR "Postpartum women" OR "Puerperium OR "Working women" OR "Working mother" OR "Working mothers" OR "Working mom" OR "Employed mother" OR "Employed mother"	3,549
Intervention	“Real-time” OR Online OR Tele-lactation OR Telelactation OR Telehealth OR Telemedicine OR “Remote lactation” OR “Mobile lactation” OR “Mobile breastfeeding” OR mHealth OR “Telephone support” OR “Telephone call” OR Helpline OR Hotline OR “Virtual lactation” OR “Virtual breastfeeding service” OR Videoconference OR “Video call”	231,200
Comparison	“Usual care” OR “Care Standard” OR “Standards Care” OR “Normal care” OR “Traditional care” OR “Routine care”	7,611
Outcomes	“Exclusive breastfeeding” OR “Any breastfeeding” OR Breastfed OR “Rate of exclusive breastfeeding” OR “Breastfeeding rate” OR “Duration of breastfeeding” OR “Breastfeeding duration” OR “Breastfeeding skills” OR “Breastfeeding efficacy” OR “Breastfeeding practice” OR “Breastfeeding Self-efficacy” OR “Breastfeeding knowledge”	2655
	P AND I AND C AND O	2

Limitation

1. Year: 2012-2022 2. Language: English or Thai
3. Articles Type: Research articles, Review articles, Cases report

Search conducted on **ScienceDirect** from 2012 to 2022 (7/06/2022)

PICO	Search Terms	Numbers of studies
Participants	"Pregnant women" OR "Postpartum mothers" OR "Postpartum women" OR "Postpartum Period" OR Puerperium OR "Lactation mothers" OR "Working mothers" OR "Employed mothers"	19,213
Intervention	“Real-time” OR Telelactation OR Telehealth OR Telemedicine OR “Mobile breastfeeding” OR mHealth OR “Virtual breastfeeding” OR Videoconference	335,553
Comparison	“Usual care” OR “Care Standard” OR “Standards Care” OR “Normal care” OR “Traditional care” OR “Routine care”	10,721
Outcomes	“Exclusive breastfeeding” OR “Any breastfeeding” OR “Breastfeeding rate” OR “Breastfeeding duration” OR “Breastfeeding skills” OR “Breastfeeding practice” OR “Breastfeeding Self-efficacy” OR “Breastfeeding knowledge”	7,686
Combined PICO with AND	("Working mothers" OR "Employed mothers") AND (Telelactation OR mHealth) AND (“Usual care” OR “Standards Care”) AND (“Breastfeeding rate” OR “Breastfeeding duration”)	1
	("Postpartum mothers" OR "Pregnant women") AND (“Real-time Telelactation” OR Telemedicine) AND (“Routine care” OR “Standard Care”) AND (“Exclusive breastfeeding” OR “Breastfeeding Self-efficacy”)	5
	("Postpartum women" OR "Employed mothers") AND (Telehealth OR Videoconference) AND (“Usual care” OR “Standards Care”) AND (Breastfeeding OR “Breastfeeding practice”)	3
	Combined	9

Limitation

1. Years: 2012-2022
2. Language: English or Thai
3. Articles Type: Research articles, Review articles
4. Access types: Open Access & Open archive

Search conducted on **Scopus** from 2012 to 2022 (8/06/2022)

PICO	Search Terms	Numbers of studies
Participants	(Pregnancy) OR ("Pregnant women") OR ("Postpartum mothers") OR (mother) OR ("Postpartum Period") OR (postpartum) OR ("Postpartum mother") OR ("Postpartum women") OR (puerperium) OR ("Working women") OR ("Working mothers") OR ("Working mom") OR ("Employed mother")	1,081,778
Intervention	(Real-time) OR (Online) OR (Tele-lactation) OR (Telelactation) OR (Telehealth) OR (Telemedicine) OR (“Remote lactation”) OR (“Mobile lactation”) OR (“Mobile breastfeeding”) OR (mHealth) OR (“Telephone support”) OR (“Telephone call”) OR (Helpline) OR (Hotline) OR (“Virtual lactation”) OR (“Virtual breastfeeding service”) OR (Videoconference) OR (“Video call”)	5,003,955
Comparison	(“Usual care”) OR (“Care Standard”) OR (“Standards Care”) OR (“Normal care”) OR (“Traditional care”) OR (“Routine care”)	85,555
Outcomes	(“Exclusive breastfeeding”) OR (“Any breastfeeding”) OR (Breastfed) OR “Exclusive breastfeeding rate”) OR (“Breastfeeding rate”) OR (“Breastfeeding duration”) OR (“Breastfeeding skill”) OR (“Breastfeeding efficacy”) OR (“Breastfeeding practice”) OR (“Breastfeeding self-efficacy”) OR (Self-efficacy) OR (“Breastfeeding knowledge”)	284,473
	P AND I AND C AND O	589

The initial keyword to be used will be: (("Pregnancy") OR ("Pregnant women") OR ("Postpartum mothers") OR ("mother") OR ("Postpartum Period") OR ("postpartum") OR ("Postpartum mother") OR ("Postpartum women") OR (puerperium) OR ("Working women") OR ("Working mothers") OR ("Working mom") OR ("Employed mother")) AND (("Real-time") OR ("Online") OR ("Tele-lactation") OR ("Telelactation") OR ("Telehealth") OR ("Telemedicine") OR ("Remote lactation") OR ("Mobile lactation") OR ("Mobile breastfeeding") OR ("mHealth") OR ("Telephone support") OR ("Telephone call") OR ("Helpline") OR ("Hotline") OR ("Virtual lactation") OR (

"Virtual breastfeeding service") OR ("Videoconference") OR ("Video call")) AND (("Usual care") OR ("Care Standard") OR ("Standards Care") OR ("Normal care") OR ("Traditional care") OR ("Routine care")) AND (("Exclusive breastfeeding") OR ("Any breastfeeding") OR ("Breastfed") OR ("Exclusive breastfeeding rate") OR ("Breastfeeding rate") OR ("Breastfeeding duration") OR ("Breastfeeding skill") OR ("Breastfeeding efficacy") OR ("Breastfeeding practice") OR ("Breastfeeding self-efficacy") OR ("Self-efficacy") OR ("Breastfeeding knowledge"))

Limitation

1. Years: 2012-2022
2. Language: English
3. Subject area: Nursing, Multidisciplinary, Health profession, Medicine
4. Document Types: Articles, Review
5. Open access: All open access

Search conducted on **SpringerLink (e-Journal)** from 2012 to 2022 (11/07/2022)

PICO	Search Terms	Numbers of studies
Participants	(Pregnant OR Pregnancy OR women OR Postpartum OR mother OR Working OR Employed)	25,529
Intervention	(Real-time OR Online OR Tele-lactation OR Telelactation OR Telehealth OR Telemedicine OR Remote lactation OR Mobile OR Breastfeeding OR mHealth OR Telephone support OR Helpline OR Hotline OR Virtual OR Service OR Videoconference OR Call)	5,575
Comparison	(Usual care OR Standard OR Normal Traditional OR Routine)	1,994
Outcomes	(Exclusive breastfeeding OR Any OR Breastfed OR Rate OR Breastfeeding OR Duration OR Skill OR Efficacy OR Practice" Self-efficacy OR Knowledge)	1,359
	P AND I AND C AND O	202

Limitation

1. Years: 2012-2022
2. Language: English
3. Content Type: Articles
4. Discipline: Medicine and Public health
5. Subdiscipline: Medicine/Public health, General, Public health

Search conducted on **CINAHL Completed** from 2012 to 2022 (13/07/2022)

PICO	Search Terms	Numbers of studies
Participants	Pregnancy OR "Pregnant women" OR "Postpartum mothers" OR Mother OR "Postpartum Period" OR Postpartum OR "Postpartum mother" OR "Postpartum women" OR "Puerperium OR "Working women" OR "Working mother" OR "Working mothers" OR "Working mom" OR "Employed mother" OR "Employed mother*"	319,689
Intervention	“Real-time” OR Online OR Tele-lactation OR Telelactation OR Telehealth OR Telemedicine OR “Remote lactation” OR “Mobile lactation” OR “Mobile breastfeeding” OR mHealth OR “Telephone support” OR “Telephone call” OR Helpline OR Hotline OR “Virtual lactation” OR “Virtual breastfeeding service*” OR Videoconference* OR “Video call”	441,972
Comparison	“Usual care” OR “Care Standard” OR “Standards Care” OR “Normal care” OR “Traditional care” OR “Routine care”	26,217
Outcomes	“Exclusive breastfeeding” OR “Any breastfeeding” OR Breastfed OR “Rate of exclusive breastfeeding” OR “Breastfeeding rate” OR “Duration of breastfeeding” OR “Breastfeeding duration” OR “Breastfeeding skill*” OR “Breastfeeding efficacy” OR “Breastfeeding practice” OR “Breastfeeding Self-efficacy” OR “Breastfeeding knowledge”	10,710
	P AND I AND C AND O	159

Limitation

1. Year: 2012-2022 2. Language: English or Thai

Search conducted on **Cochrane Library (PICO Search)** from 2012 to 2022 (24/06/2022)

PICO	Search Terms	Numbers of studies
Participants	Employee OR Postpartum	0
Intervention	Telemedicine OR Telephone counseling	
Comparison	Usual care	
Outcomes	Exclusive breastfeeding	

Search conducted on **Cochrane Library (Search and MeSH)** from 2012 to 2022 (13/07/2022)

PICO	Search Terms	Numbers of studies
Participants	Pregnancy, Pregnant women, Postpartum mothers, working women, working mothers, Employed mother	9
Intervention	Real-time, Online, Tele-lactation, Telelactation, Telehealth, Telemedicine, Remote lactation, Mobile lactation, Mobile breastfeeding, mHealth, Telephone support, Telephone call, Helpline, Hotline, Virtual lactation, Virtual breastfeeding service, Videoconference, Video call	0
Comparison	Usual care, Care Standard, Standards Care, Normal care, Traditional care, Routine care	25
Outcomes	Exclusive breastfeeding, any breastfeeding, Breastfed, Rate of exclusive breastfeeding, Breastfeeding rate, Duration of breastfeeding, Breastfeeding duration, Breastfeeding skill, breastfeeding efficacy, breastfeeding practice, Breastfeeding Self-efficacy, Breastfeeding knowledge	0
Combination with AND	P And I AND C AND O	0
Keyword Breastfeeding	- Cochrane Review<br>- Trials; Source: PubMed<br>: Embase<br>: CNAHL	56<br>669<br>823<br>1
	Cochrane Review<br>Trials; Embase	56<br>823

*From the results, we will export 823 studies from Embase database because we got the articles from PubMed and CINAHL already.

Limitation 1. Year: 2012-2022 2. Language: English or Thai

Search conducted on **WHO International Clinical Trials Registry Platform (ICTRP)** (14/0672022)

PICO	Search Terms	Numbers of studies
Participants	Employee OR Postpartum	0
Intervention	Telemedicine OR Telephone counseling	
Comparison	Usual care	
Outcomes	Exclusive breastfeeding	

Search conducted on **Thai Journals Online (ThaiJO)** from 2012 to 2022 (17/06/2022)

PICO	Search Terms	Numbers of studies
Participants	หญิงตั้งครรภ์ที่จะเลี้ยงลูกด้วยนมแม่ หรือ<br>มารดาหลังคลอด หรือ<br>มารดาทำงานนอกบ้าน หรือ<br>มารดาให้นมบุตร	10<br>714<br>21<br>175
Intervention	โปรแกรมส่งเสริมการเลี้ยงลูกด้วยนมแม่<br>การให้ข้อมูลทางออนไลน์หรือโซเชียลมีเดีย<br>การให้คำแนะนำทางโทรศัพท์	61<br>16<br>61
Comparison	-	-
Outcomes	อัตราการเลี้ยงลูกด้วยนมแม่อย่างเดียว 6 เดือน<br>การเลี้ยงลูกด้วยนมมารดา 6 เดือน	44<br>89
Combination	Manual screening	40

Limitation

1. Years 2012-2022
2. Language: Thai or English

Search conducted on **Google Scholar** (30/06/2022)

PICO	Search Terms	Numbers of studies
Participants	Employed mothers	141
Intervention	Telelactation	
Comparison	-	
Outcomes	Exclusive breastfeeding	

The initial keyword will be “Telelactation on exclusive breastfeeding in employed mothers” on Google Scholar basic search.

Search conducted by References list (1/08/2022 to 30/09/2022) from an existing systematic review of Tele-lactation or Telephone support or mHealth or Mobile health or Videoconference in breastfeeding = 60 studies

Update searched June 28, 2023

The total number of studies exported to Endnote20 is as follows,

Data based: 34+52+2+9+589+202+159+56+823= 1,926 records

Other method: 40+141+60 = 241 studies

Total = 2,167 studies
