# Supplementary material for: The effectiveness of real-time telelactation intervention on breastfeeding outcomes among employed mothers: a systematic review and meta-analysis
Source: BMC Pregnancy Childbirth. 2025 Mar 25;25:341. doi: 10.1186/s12884-025-07440-3 (PMC11934809; doi:10.1186/s12884-025-07440-3)
Supplement: Supplementary file 2 — Supplementary Material 2 [file 12884_2025_7440_MOESM2_ESM.pdf]

Supplementary 2 Subgroup analysis for testing the heterogeneity

Outcomes	Factors	Groups	RR (95%CI)	*P* value	Heterogeneity (%)	*P* value for heterogeneity	*P* value between subgroups
EBF at 5-6 months	Timing of the intervention Start	Pregnant women [47, 48, 53, 57, 59, 62, 63]	1.64 (1.20-2.23)	0.002	37	0.150	0.03
		Postpartum women [49-51, 54, 60, 61]	1.12 (0.97-1.31)	0.013	63	0.020	
	Providers	Healthcare [47, 50, 51, 53, 54, 57, 59, 61, 63]	1.57 (1.28-1.93)	<0.0001	11	0.35	0.004
		Non-healthcare [48, 49]	1.11 (0.98-1.25)	0.10	0	0.90	
	Service models	Proactive [47, 48, 50, 51, 57, 59, 63]	2.23 (1.26-3.94)	0.006	0	<0.0001	0.0002
		Reactive [60, 62]	0.98 (0.93-1.04)	0.54	0	0.42	
		Mixed [49, 53, 54, 61]	1.38 (1.01-1.87)	0.04	63	0.04	

Note. EBF, Exclusive breastfeeding—the infant receives only breast milk, no other foods, liquids, or water (except for medications, vitamin or mineral supplements); **RR,** Relative risk; **CI,** Confidence interval.
